# Immunoprotective effects of extracellular products of *Pasteurella multocida* on mice

**DOI:** 10.3389/fmicb.2025.1674831

**Published:** 2025-09-09

**Authors:** Jingtao Li, Wan Liu, Xiaoyu Zhang, Yang Song, Li Chen, Qiumei Shi, Tonglei Wu

**Affiliations:** ^1^Hebei Provincial Key Laboratory of Preventive Veterinary Medicine, Hebei Normal University of Science and Technology, Qinhuangdao, China; ^2^Elanco (Sichuan) Animal Health Co., Ltd., Chengdu, China

**Keywords:** *Pasteurella multocida*, extracellular products, vaccine, immune response, cross-protection

## Abstract

**Background:**

*Pasteurella multocida* (*P. multocida*) is a globally significant pathogen causing severe infections in livestock, including hemorrhagic septicemia and respiratory diseases. Current vaccines offer limited serotype-specific protection, particularly against serotype A:3, a major cause of bovine respiratory disease. Extracellular products (ECPs) of bacteria, containing secreted proteins and enzymes, have shown promise as immunogens in other pathogens, but their potential against *P. multocida* remains unclear.

**Methods:**

Extracellular products were isolated from *P. multocida* serotype A:3 strain PmQA-1 and characterized via SDS-PAGE, mass spectrometry, and enzymatic activity assays. Pathogenicity was evaluated by determining the median lethal dose (LD_50_) in mice. Mice were immunized with ECPs, formalin-killed cells (FKC), or a combination (FKC + ECPs), and immune responses (serum IgG, splenic lymphocyte proliferation, cytokine expression) were assessed over 28 days. Protective efficacy was tested via challenge with homologous (A:3) and heterologous (B:2, D:4) strains.

**Results:**

Extracellular products contained 157 proteins (25–100 kDa), including immunogenic factors like transferrin-binding protein A, and exhibited stable amylase activity. The LD_50_ of ECPs in mice was 2.69 mg/mouse, inducing lesions typical of *P. multocida* infection. ECP-immunized mice showed peak IgG levels at day 21, enhanced lymphocyte proliferation, and upregulated TNF-α, IFN-γ, IL-1β, and IL-10 in key tissues. Challenge experiments demonstrated 100% survival against A:3 and B:2, and 90% against D:4, outperforming FKC and FKC + ECPs.

**Conclusion:**

Extracellular products from *P. multocida* serotype A:3 induce robust humoral and cellular immunity, providing broad-spectrum protection against multiple serotypes. These findings support ECPs as a promising subunit vaccine candidate for controlling *P. multocida* infections in livestock.

## 1 Introduction

*P. multocida* is a Gram-negative bacterium commonly colonizing the upper respiratory mucosa (oropharyngeal and nasopharyngeal) of a wide range of wild and domesticated animals, including species relevant to the livestock and food production industries such as cattle, swine, poultry, rabbits, and even humans (Piorunek et al., 2023; Ryan and Feder, 2019; Wilkie et al., 2012). Under stress-inducing conditions, this typically commensal bacterium may invade host tissues, resulting in systemic or localized infections such as pneumonia, hemorrhagic septicemia (HS), atrophic rhinitis, avian cholera, cellulitis, dermonecrosis, abscesses, and meningitis (Dassanayake et al., 2025; Zhao et al., 2024).

Based on capsular composition, *P. multocida* is classified into five capsular serogroups (A, B, D, E, and F), and based on differences in lipopolysaccharide (LPS) structure, into 16 somatic serotypes (Guan et al., 2020; Gulliver et al., 2022; Megroz et al., 2016). These serogroups and serotypes differ in host specificity and disease manifestation. For instance, in cattle, *P. multocida* causes two major syndromes: hemorrhagic septicemia (HS), primarily associated with serogroup B:2 strains, and bovine respiratory disease complex (BRD), primarily caused by serogroup A:3 strains. HS is a rapidly progressing, frequently fatal septicemia, especially prevalent in Africa and Asia, with significant impact on smallholder farms. In contrast, BRD (or “shipping fever”) is a multifactorial disease affecting the upper or lower respiratory tract, typically occurring in recently weaned or transported calves (Zhan et al., 2021). In China, *P. multocida* serotype D:4 strains are also frequently associated with cattle pneumonia (Sun et al., 2022).

Despite decades of vaccine deployment, *P. multocida* infections remain a persistent challenge in cattle and other livestock. Current vaccines-including inactivated whole-cell (bacterin), live attenuated, and subunit formulations-exhibit notable limitations in terms of safety, efficacy, and duration of protection. Bacterin vaccines typically confer only 4–6 months of immunity (Varshney et al., 2020), while live attenuated vaccines-mainly derived from serogroup B strains-may extend protection up to 1 year. However, both types of vaccines offer limited, serogroup-specific protection and fail to provide adequate immunity against *P. multocida* serotype A:3, the predominant strain associated with bovine respiratory disease. In recent years, substantial efforts have been made to identify alternative subunit vaccine candidates. Targets under investigation include capsular polysaccharides, lipopolysaccharides (LPS), siderophores, *P. multocida* toxin (PMT), dermonecrotic toxins, and various outer membrane proteins (OMPs) (Dabo et al., 2008; Liao et al., 2006; Pettit et al., 1993; Zhao X. et al., 2022). Among these, PlpE-a conserved, surface-exposed protein-has shown particular promise. Hatfaludi et al. (2012) identified over 70 putative surface or secreted proteins through bioinformatic analyses, but only recombinant PlpE demonstrated complete protection in challenge models. Subsequent studies reported partial protection using recombinant OmpH, PlpE, and PlpEC-OmpH fusion proteins, achieving protection rates of up to 80% (Okay et al., 2012). Furthermore, a recently identified surface lipoprotein, PmSLP, has demonstrated strong protective efficacy (75%–87.5% survival) in cattle challenged with virulent *Glaesserella parasuis (G. parasuis)* strains (Nguyen et al., 2025). Extracellular products (ECPs) are secreted metabolites produced by bacteria during growth, including various enzymes and toxins such as proteases, amylases, lipases, lecithinases, gelatinases, ureases, chitinases, DNases, and hemolysins. These factors contribute significantly to bacterial virulence by degrading host tissues, evading immune responses, and facilitating colonization (Baldissera et al., 2018; Pemberton et al., 1997; Sahu et al., 2011; Zhao Y. et al., 2022). In other pathogens-such as *Aeromonas veronii (A. veronii)*, *Vibrio vulnificus (V. vulnificus)*, and *Moritella viscosa (M. viscosa)*-ECPs have demonstrated strong immunogenicity and provided effective protection in animal models (Lee et al., 1997; MacKinnon et al., 2019; Song et al., 2018).

Given this potential, we focused on a bovine-origin *P. multocida* serotype A:3 strain (PmQA-1), commonly associated with BRD in cattle. We prepared its ECPs, evaluated enzymatic activities and pathogenicity in mice, and formulated an ECP-based subunit vaccine. Using a murine infection model, we assessed its protective efficacy against different *P. multocida* serotypes. Our results showed that although the vaccine based on ECPs provided limited cross-protection against strains of other serotypes, it granted complete (100%) protection against the challenge of homologous serotype A:3, which emphasized its potential as a candidate for a novel subunit vaccine.

## 2 Materials and methods

### 2.1 Bacterial strains

The strains PmQA-1 (serotype A:3), PmQB-1 (serotype B:2), and PmQD-1 (serotype D:4) were used in this study. All strains were cultured overnight at 37 °C in tryptic soy broth (TSB; Qingdao Hope Bio-Technology Co., Ltd., Qingdao, China) or on tryptic soy agar (TSA; Qingdao Hope Bio-Technology Co., Ltd., Qingdao, China), supplemented with 10% fetal bovine serum (FBS, Zhejiang Tianhang Biotechnology Co., Ltd., Hangzhou, China) and NAD (Beijing Bio-Lab Biotechnology Co., Ltd., Beijing, China).

### 2.2 Experimental animals and ethical statement

KM mice (6–8 weeks old) were purchased from SPF (Beijing, China) Biotechnology Co., Ltd. Throughout the experiment, the mice were housed under sterile conditions in individually ventilated cages, with an ambient temperature maintained at 22.0 °C ± 0.5 °C and relative humidity at 60% ± 10%. A 12-h light/dark cycle was applied. All animal procedures were conducted in strict accordance with the Experimental Animal Welfare and Ethical Regulations issued by the Hebei Provincial Department of Science and Technology (HPDST 2020-17).

### 2.3 Preparation of ECPs from *P. multocida* PmQA-1

A single colony of *P. multocida* PmQA-1 was inoculated into 2 L of TSB and incubated at 37 °C with shaking at 180 rpm for 36 h. The culture was then centrifuged at 10,000 rpm for 20 min at 4 °C to collect the supernatant. The obtained supernatant was sterilized by filtration through a 0.22 μm disposable syringe filter (Merck Millipore Ltd., USA). To precipitate the extracellular proteins, ammonium sulfate was added to achieve 85% saturation and the solution was kept at 4 °C overnight. The precipitated proteins were recovered by centrifugation at 10,000 rpm for 20 min at 4 °C, and the supernatant was discarded. The resulting pellet was resuspended in 0.02 mol/L Tris-HCl buffer (pH 7.5) and dialyzed against the same buffer using dialysis tubing with a molecular weight cut-off of 3,500 Da (Beijing Solarbio Science & Technology Co., Ltd., Beijing, China). After dialysis, the ECP solution was concentrated to a final volume of 15 mL using PEG-20000 at 4 °C, and sterilized by filtration through a 0.22 μm syringe filter. To ensure sterility, an aliquot of the ECP preparation was spread onto TSA plates and incubated overnight at 37 °C. Sterile preparations were aliquoted and stored at −80 °C until use.

The total protein concentration of the ECPs was determined using a BCA Protein Assay Kit (Beijing Solarbio Science & Technology Co., Ltd., Beijing, China) following the manufacturer’s instructions. Briefly, the BCA working solution was prepared by mixing BCA reagent with Cu reagent at a volume ratio of 50:1. A bovine serum albumin (BSA) standard was prepared by diluting 10 μL of stock solution with PBS to a final volume of 100 μL to yield a concentration of 0.5 mg/mL. Serial volumes (0, 2, 4, 6, 8, 12, 16, and 20 μL) of the BSA standard were added to a 96-well microplate and adjusted to 20 μL per well with PBS. ECP samples were two-fold serially diluted, and 20 μL of each dilution was added to the sample wells. Then, 200 μL of BCA working solution was added to each well and incubated at 37 °C for 15–30 min. Absorbance at 562 nm was measured using a microplate reader, and the total protein concentration was calculated based on the standard curve.

### 2.4 SDS-PAGE and mass spectrometry analysis of ECPs

The ECP samples were mixed with protein loading buffer and boiled for 10 min. After cooling, the samples were subjected to SDS-PAGE. Upon completion of electrophoresis, the gels were stained, destained, and photographed for record-keeping. For proteomic profiling, the ECP samples were sent to Shanghai Majorbio Bio-Pharm Technology Co., Ltd., for LC-MS/MS analysis. In brief, the ECPs were first quality-checked, then reduced and alkylated, followed by trypsin digestion. The resulting peptides were quantified, and equal amounts were analyzed by LC-MS/MS. The acquired spectra were searched using the Sequest or Mascot algorithm in Proteome Discoverer, and the identified proteins were subjected to statistical analysis.

### 2.5 Enzymatic activity assays of ECPs

#### 2.5.1 Amylase activity

Tryptone Soy Agar (TSA) plates containing 2% soluble starch were prepared and sterilized by autoclaving. Wells were punched into the solidified medium, and the plates were overlaid with 0.5% sterile agar. Subsequently, 40 μL of ECPs were added to each well and incubated at 37 °C for 48 h. After incubation, iodine solution was applied around the wells. The presence of a clear hydrolytic zone indicated a positive result, while no color change indicated a negative result.

#### 2.5.2 Urease activity

TSA medium containing 0.2% phenol red indicator was sterilized, and a sterile urea solution was added to a final concentration of 2% before pouring the plates. Wells were made and overlaid with 0.5% sterile agar. Then, 40 μL of ECPs were added to each well and incubated at 37 °C for 48 h. A pink halo around the wells indicated urease activity, while the absence of a color change indicated a negative result.

#### 2.5.3 Protease activity

Sterilized TSA medium was supplemented with 8% skimmed milk powder and poured into plates. Wells were punched, overlaid with 0.5% sterile agar, and 40 μL of ECPs were added to each well. After incubation at 37 °C for 48 h, 10% trichloroacetic acid was dropped around the wells to stop the reaction. A clear hydrolytic zone indicated protease activity; no change indicated a negative result.

#### 2.5.4 Lecithinase activity

TSA medium was sterilized and supplemented with 1% fresh SPF chicken egg yolk emulsion before pouring the plates. Wells were prepared and overlaid with 0.5% sterile agar. Then, 40 μL of ECPs were added and incubated at 37 °C for 48 h. The formation of an opaque, milky-white halo indicated positive phospholipase activity; absence of such a zone indicated a negative result.

#### 2.5.5 Lipase activity

TSA plates containing 1% Tween-80 were prepared. Wells were punched and overlaid with 0.5% sterile agar, and 40 μL of ECPs were added to each well. After incubation at 37 °C for 48 h, a white, opaque halo indicated lipase activity; no change indicated a negative result.

#### 2.5.6 Gelatinase activity

TSA medium containing 0.4% gelatin was sterilized and poured into plates. Wells were made and overlaid with 0.5% sterile agar. Then, 40 μL of ECPs were added and incubated at 37 °C for 48 h. A clear hydrolytic zone indicated gelatinase activity; absence of a zone indicated a negative result.

#### 2.5.7 Hemolysin activity

Wells were prepared in blood agar plates (Beijing Land Bridge Technology Co., Ltd., Beijing, China) and overlaid with 0.5% sterile agar. 40 μL of ECPs were added to each well and incubated at 37 °C for 48 h. A clear, colorless hemolytic zone around the wells indicated hemolysin activity; no change indicated a negative result.

### 2.6 Effects of environmental physicochemical factors on ECPs enzymatic activity

A single sterile colony of *P. multocida* PmQA-1 was inoculated into 500 mL of TSB and cultured at 37 °C with shaking at 180 rpm. To assess the effect of culture duration on enzymatic activity, 100 mL of culture was collected at 12 h, 24 h, 36 h, 48 h, and 60 h, respectively, and ECPs were prepared as described above.

To investigate the effect of temperature on ECP enzymatic activity, aliquots of the prepared ECPs were equally divided into five groups (2 mL per group). The first three groups were incubated in water baths at 20 °C, 30 °C, and 37 °C for 24 h, respectively. The fourth group was treated at 60 °C for 2 h, and the fifth group at 80 °C for 1 h. After treatment, all samples were immediately cooled on ice, and enzyme activities were measured.

To examine the effect of pH, the prepared ECPs were also equally divided into five groups (2 mL per group). The pH of each group was adjusted to 3, 5, 7, 9, or 11 using 1 mol/L HCl or 1 mol/L NaOH. Samples were maintained at room temperature for 1 h, after which the pH of all samples was readjusted to neutral (pH 7) prior to enzymatic activity assays.

### 2.7 Determination of LD_50_ for *P. multocida* and its ECPs

Single colonies of *P. multocida* strains PmQA-1, PmQB-1, and PmQD-1 were inoculated into TSB and cultured to the logarithmic growth phase. The bacterial cells were collected, washed with PBS, serially diluted, and plated for colony counting. Aliquots were prepared and stored at −80 °C until use.

A total of 80 female KM mice (6–8 weeks old) were randomly divided into 16 groups (*n* = 5 per group). Based on viable counts, groups 1–5 were intraperitoneally injected with 0.2 mL of PmQA-1 suspensions at concentrations of 1.95 × 10^5^, 1.95 × 10^4^, 1.95 × 10^3^, 1.95 × 10^2^, and 1.95 × 10^1^ CFU/mL, respectively. Groups 6–10 received PmQB-1 at 4.87 × 10^5^, 4.87 × 10^4^, 4.87 × 10^3^, 4.87 × 10^2^, and 4.87 × 10^1^ CFU/mL, respectively. Groups 11–15 were injected with PmQD-1 at concentrations of 1.24 × 10^5^, 1.24 × 10^4^, 1.24 × 10^3^, 1.24 × 10^2^, and 1.24 × 10^1^ CFU/mL, respectively. Group 16 served as the negative control and received 0.2 mL of PBS.

For ECPs toxicity determination, 30 female KM mice (6–8 weeks old) were randomly assigned into six groups (*n* = 5 per group). Groups 1–5 were intraperitoneally injected with 0.2 mL of PmQA-1 ECPs at concentrations of 20.42, 10.21, 5.11, 2.55, and 1.28 mg/mL, respectively. Group 6 served as the control and received 0.2 mL of sterile PBS.

All mice were observed for clinical symptoms and mortality for seven days after injection. The median lethal dose (LD_50_) was calculated using the modified Kärber method as follows:

LD_50_ = log-1{X_*k*_- i[p-(3-P_*m*_-P_*n*_)/4]}

where i is the logarithmic interval between consecutive doses, X_*k*_ is the logarithm of the highest dose, p is the sum of the mortality proportions for each dose group, P_*m*_ is the highest mortality proportion, and P_*n*_ is the lowest mortality proportion.

### 2.8 Preparation of vaccines

Based on the method described by reference (Yuan et al., 2022) and the LD_50_ results for ECPs, the ECPs were diluted to a final concentration of 13.45 mg/mL. Equal volumes of the diluted ECPs and Freund’s complete adjuvant (Sigma-Aldrich Co., LLC, Missouri, USA) were thoroughly mixed and emulsified. To confirm successful emulsification, a drop of the mixture was added to water; if the droplet remained intact without dispersing, the ECP vaccine was deemed ready for use.

For the preparation of the inactivated *P. multocida* vaccine (FKC), a bacterial suspension of PmQA-1 at a concentration of 1 × 10^7^ CFU/mL was mixed with formaldehyde to a final concentration of 0.4% and incubated at 4 °C for 24 h to achieve inactivation. The suspension was then centrifuged at 6,500 rpm for 5 min, and the supernatant was discarded. The bacterial pellet was washed three times with PBS to remove residual formaldehyde and resuspended in PBS to the original volume. Sterility was confirmed by plating an aliquot onto TSA and incubating overnight at 37 °C; absence of colony growth indicated complete inactivation. The inactivated bacterial suspension was then emulsified with an equal volume of Freund’s complete adjuvant to prepare the final FKC vaccine.

To prepare the combined vaccine (FKC + ECPs), equal volumes of the ECP vaccine and the FKC vaccine were mixed thoroughly to form a homogenous emulsion.

### 2.9 Immunization and sample collection in mice

Twenty female KM mice (6–8 weeks old) were randomly divided into four groups (*n* = 5 per group) to determine the optimal safe dose for vaccination. Mice were subcutaneously immunized at multiple sites with the FKC vaccine at doses of 0.6 mL, 0.4 mL, 0.2 mL, and 0.1 mL, respectively. The animals were monitored for general health and local skin reactions. The highest safe dose was selected for subsequent immunization experiments.

For the main immunization trial, 216 female KM mice (6–8 weeks old) were randomly assigned to four groups (*n* = 54 per group). Immunization was performed subcutaneously at multiple sites following the procedure recommended for commercial *P. multocida* vaccines. The groups were as follows: the ECPs group received 0.2 mL of ECP vaccine; the FKC group received 0.2 mL of FKC vaccine; the FKC + ECPs group received 0.2 mL of the combined FKC + ECPs vaccine; the PBS group received 0.2 mL of sterile PBS as a control. The first immunization was designated as day 0. On day 14, a booster immunization was administered, in which the concentrations of ECPs protein and bacteria were reduced by half compared to the initial dose, while the injection volume remained the same. The booster vaccines were fully emulsified with Freund’s incomplete adjuvant prior to administration.

On days 0, 7, 14, 21, and 28, three mice from each group were randomly selected for sample collection. Blood was collected by orbital enucleation, and sera were separated for cytokine assays. Liver, spleen, and lung tissues were also harvested for further analyses. Additionally, on days 0, 14, and 28, spleens from three randomly selected mice per group were collected for lymphocyte proliferation assays. The remaining 30 mice in each group were retained for subsequent challenge protection experiments.

### 2.10 Analysis of humoral and cellular immune responses

To prepare whole-cell proteins of PmQA-1, bacteria were cultured to the logarithmic phase and harvested by centrifugation at 12,000 rpm for 10 min. The pellet was washed three times with PBS and resuspended. Bacterial cells were disrupted using an ultrasonic homogenizer (Ningbo Scientz Biotechnology Co., Ltd., Ningbo, China) with cycles of 3 s on and 3 s off until the suspension became clear. The lysate was centrifuged at 12,000 rpm for 10 min, and the supernatant was sterilized through a 0.22 μm disposable syringe filter to obtain the whole-cell protein. The protein concentration was determined using a BCA Protein Assay Kit.

The serum IgG level was measured by indirect ELISA (iELISA). Briefly, 96-well microplates were coated with PmQA-1 whole-cell proteins at a concentration of 1000 μg/mL in carbonate buffer (0.1 mol/L, pH 9.6) with 100 μL per well and incubated at 37 °C for 4 h. Plates were washed three times with 3 mL PBS, then blocked with PBST containing 5% skimmed milk at 37 °C for 2 h. After washing, mouse sera (primary antibody) were diluted 1:50 in PBST and added at 100 μL per well, followed by incubation at 37 °C for 1 h. Plates were washed three times, then incubated with HRP-conjugated goat anti-mouse IgG (secondary antibody) diluted 1:10,000 in PBST at 100 μL per well at 37 °C for 1 h. After a final wash, 100 μL of TMB substrate was added to each well and the reaction was developed at 37 °C in the dark for 10 min. The reaction was stopped with 50 μL of 2 mol/L H_2_SO_4_, and absorbance was measured at 450 nm.

Lymphocyte proliferation in the spleen was assessed using the MTT assay. Mouse spleens were placed on 70 μm cell strainers and gently ground in 3 mL RPMI 1640 medium (Thermo Fisher Scientific Inc., New York, USA) to obtain single-cell suspensions. Cells were centrifuged at 1,000 rpm for 5 min, the supernatant was discarded, and the pellet was resuspended in 3 mL red blood cell lysis buffer for 10–15 min. After lysis, cells were washed three times with 5 mL RPMI 1640 and finally resuspended in complete RPMI 1640 medium supplemented with 10% FBS and 1% non-essential amino acids, adjusting the cell concentration to 1 × 10^7^ CFU/mL. A total of 100 μL of cell suspension was added to each well of a 96-well culture plate, together with PmQA-1 whole-cell protein at a final concentration of 5 μg per well. Cells were incubated in a CO_2_ incubator for 48 h. Then, 10 μL of MTT stock solution (5 mg/mL) was added per well, mixed gently, and incubated for another 4 h. After incubation, 100 μL of Formazan solvent was added per well, and plates were incubated for an additional 4 h with intermittent shaking to fully dissolve the crystals. Absorbance was measured at 570 nm using a microplate reader. The stimulation index (SI) was calculated as:

SI = (OD_570_ of protein-stimulated group - OD_570_ of medium)/(OD_570_ of non-stimulated group – OD_570_ of medium).

Gene expression levels of TNF-α, IFN-γ, IL-1β, and IL-10 in liver, spleen, and lung tissues were determined by quantitative PCR (qPCR). Total RNA was extracted using the RNAprep Pure Tissue Kit (TianGen Biotech Co., Ltd., Beijing, China), treated to remove genomic DNA, and reverse transcribed into cDNA. qPCR was performed using SYBR Green PCR Master Mix. The primer sequences are listed in Table 1. Cytokine expression levels were normalized to the internal control GAPDH and β-Actin genes and calculated using the 2^−ΔΔ^*^CT^* method.

**TABLE 1:** Primers used for qPCR.

Gene	Primer sequence (5′− 3′)
GAPDH	AGGTCGGTGTGAACGGATTTG
	TGTAGACCATGTAGTTGAGGTCA
β-Actin	TTCAACACCCCAGCCATG
	CCTCGTAGATGGGCACAGT
TNF-α	CCCTCACACTCAGATCATCTTCT
	GCTACGACGTGGGCTACAG
IFN-γ	ATGAACGCTACACACTGCATC
	CCATCCTTTTGCCAGTTCCTC
IL-10	CTTACTGACTGGCATGAGGATCA
	GCAGCTCTAGGAGCATGTGG
IL-1β	GACTGTTTCTAATGCCTTCCC
	ATGGTTTCTTGTGACCCTGA

### 2.11 Challenge test in immunized mice

At 28 days post-immunization, a total of 21 mice from each vaccine group (ECPs, FKC, and FKC + ECPs) and the PBS control group were randomly divided into three subgroups (*n* = 10 per subgroup). The mice were then intraperitoneally challenged with PmQA-1 (77.70 CFU/mouse), PmQB-1 (43.35 CFU/mouse), or PmQD-1 (4.38 × 10^3^ CFU/mouse). The mice were monitored daily for 14 consecutive days, and mortality was recorded for each group. The relative percent survival (RPS) was calculated using the following formula:

RPS (%) = [1 - (mortality in immunized group/mortality in control group)] × 100%

### 2.12 Statistical analysis

Statistical analyses were conducted using SPSS version 26.0 and GraphPad Prism version 9.5.0. One-way analysis of variance (ANOVA) followed by pairwise *t*-tests was applied where appropriate. Data are presented as the mean ± standard error of the mean (SEM). Statistical significance was indicated as follows: *p < 0.05, **p < 0.01, and ***p < 0.001.

## 3 Results

### 3.1 SDS-PAGE and mass spectrometry analysis of ECPs

**FIGURE 1:**
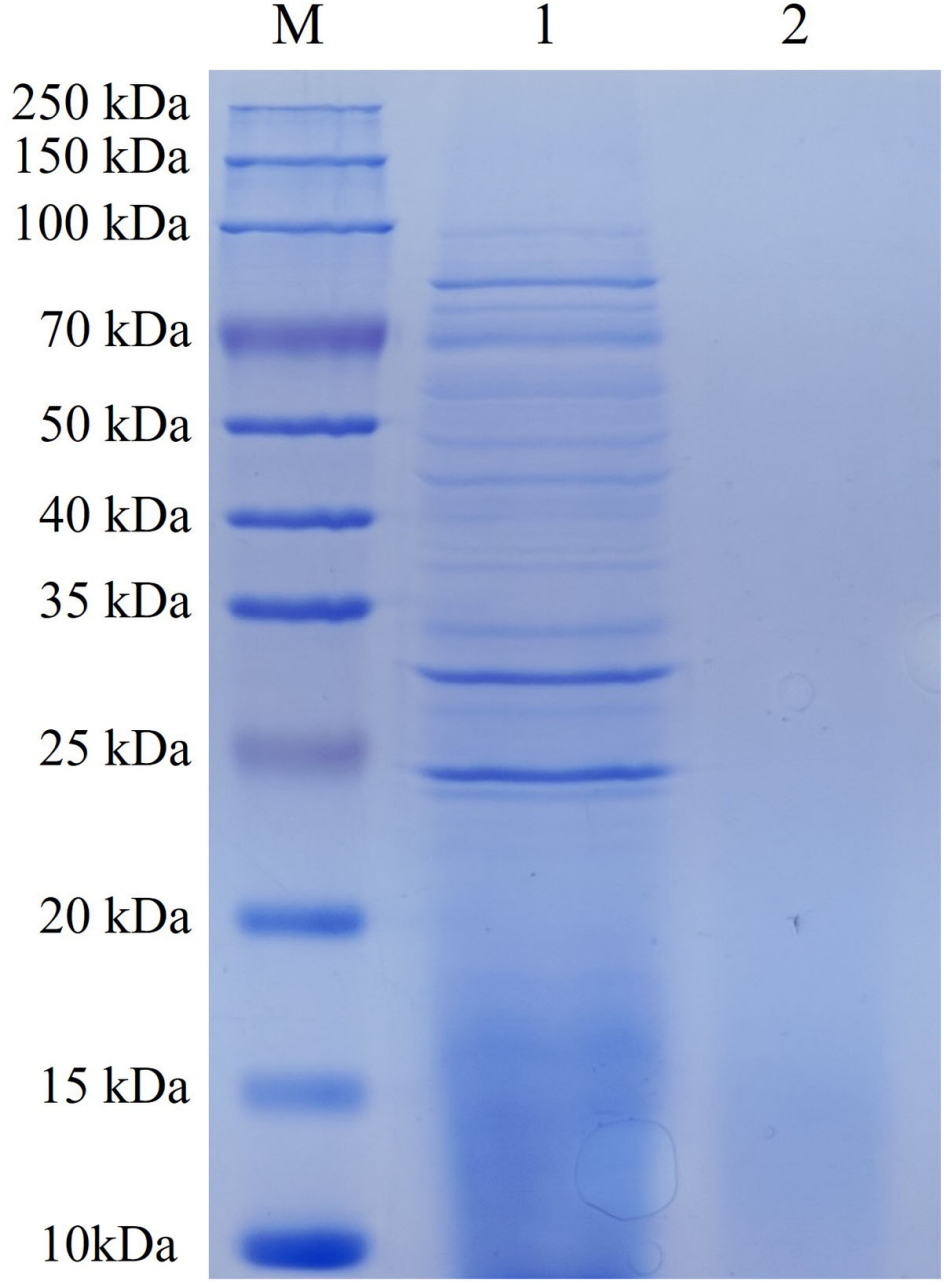
SDS-PAGE of ECPs. M, Pre-stained protein marker; 1, ECPs; 2, negative control.

The protein concentration of the prepared ECPs was determined to be 20.42 g/L using the BCA method. SDS-PAGE analysis (Figure 1) revealed that the protein bands were mainly distributed between 25 and 100 kDa. Mass spectrometry analysis (Supplementary material) identified a total of 157 proteins within the ECPs, including transferrin-binding protein A, elongation factor Tu, dihydrolipoyl dehydrogenase, pyruvate dehydrogenase E1 component, heme-binding protein A, MltA-interacting MipA protein, and metalloprotease PmbA, among others.

### 3.2 Amylase activity of ECPs

**FIGURE 2:**
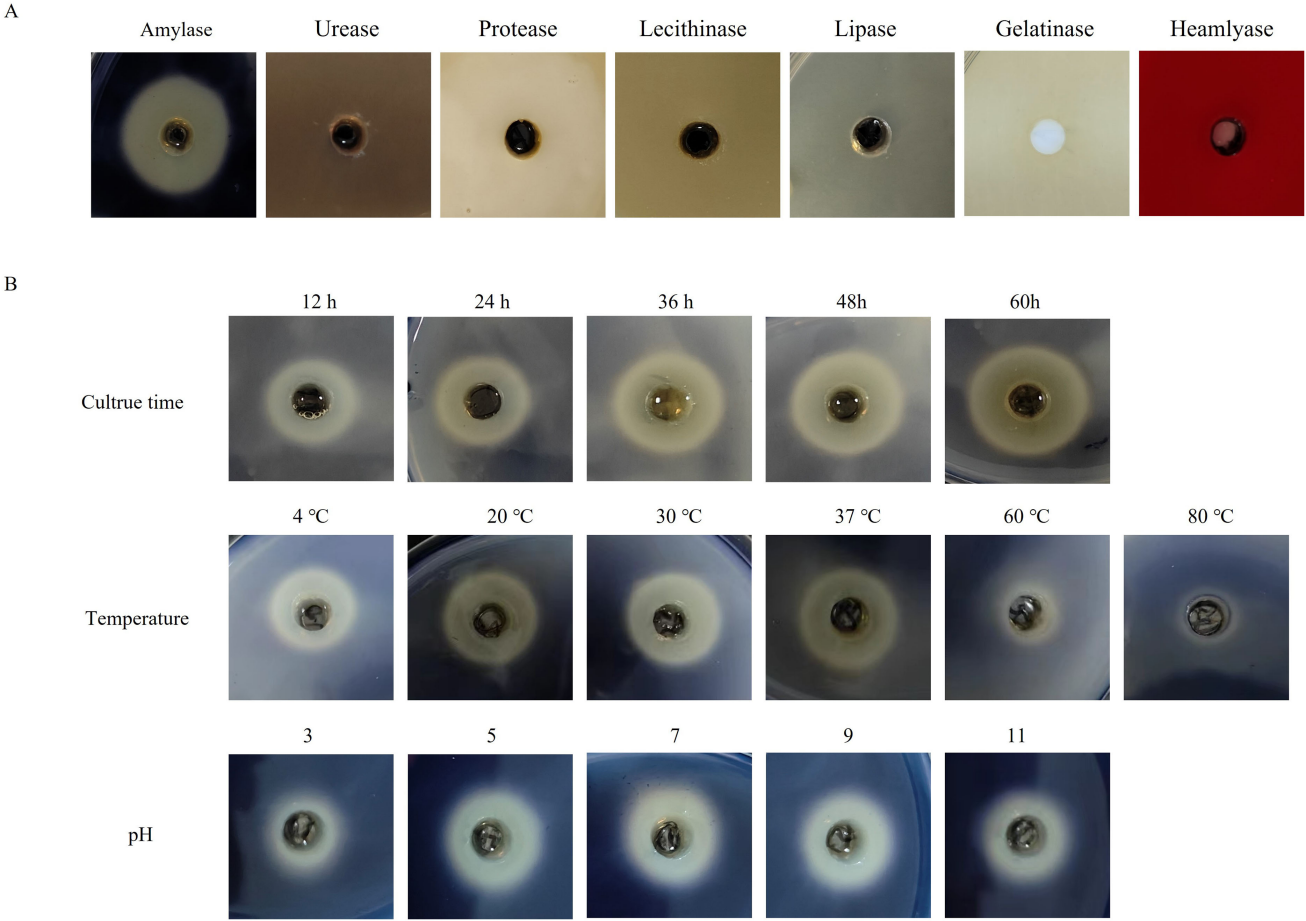
Enzymatic activity analysis of ECPs. **(A)** The prepared ECPs were applied to agar plates for the detection of amylase, urease, protease, phospholipase, lipase, gelatinase, and hemolysin activities. Plates were incubated at 37 °C for 48 h and then examined. **(B)** Effects of different culture times, temperatures, and pH values on the amylase activity of ECPs.

The enzymatic activity of the ECPs was assessed using the agar plate diffusion method. As shown in Figure 2, the ECPs exhibited amylase activity, forming clear hydrolysis zones on the agar plates, while no urease, protease, phospholipase, lipase, gelatinase, or hemolysin activities were detected.

Given the presence of amylase activity, the effects of incubation time, temperature, and pH on the amylase activity of ECPs were further investigated. As illustrated in Figure 2, amylase activity increased progressively with prolonged incubation time. Temperatures of 4 °C, 20 °C, 30 °C, and 37 °C had little effect on amylase activity; however, treatment at 60 °C resulted in reduced activity, and complete loss of activity was observed at 80 °C. Regarding pH, amylase activity increased with rising pH from 3 to 7, but showed a slight decrease as the pH increased from 7 to 11.

### 3.3 Determination of the LD_50_ of PmQA-1, PmQB-1, PmQD-1, and ECPs

To determine the immunization dose of ECPs and the challenge doses of *P. multocida*, mice were intraperitoneally injected with ECPs, PmQA-1, PmQB-1, and PmQD-1 to calculate their LD_50_ values. Following injection, mice in all groups except the PBS control group exhibited varying degrees of clinical symptoms, such as ruffled fur and lethargy. Necropsy of the deceased mice revealed pulmonary hemorrhage as well as splenomegaly and hepatomegaly with hemorrhage. Based on the modified Kärber method (Supplementary Table 1), the LD_50_ of ECPs was determined to be 2.69 mg/mouse, while the LD_50_ values for PmQA-1, PmQB-1, and PmQD-1 were 15.50 CFU/mouse, 8.67 CFU/mouse, and 8.76 × 10^2^ CFU/mouse, respectively.

Accordingly, for immunization, a dose equal to half of the ECPs LD_50_ (1.35 mg/mouse) was used for subcutaneous injection. For the challenge test, a dose equivalent to five times the bacterial LD_50_ was administered intraperitoneally, resulting in final challenge doses of 77.70 CFU/mouse for PmQA-1, 43.35 CFU/mouse for PmQB-1, and 4.38 × 10^3^ CFU/mouse for PmQD-1.

### 3.4 Specific IgG following immunization

Mice were immunized with different doses of the FKC and ECPs + FKC vaccine. When administered at doses of 0.6 mL and 0.4 mL, the mice exhibited noticeable adverse reactions, including lethargy and ruffled fur. Additionally, mice receiving the 0.6 mL dose developed subcutaneous abscesses and ulcers. In contrast, no obvious abnormalities were observed in mice immunized with 0.2 mL or 0.1 mL doses. Therefore, a dose of 0.2 mL FKC and ECPs + FKC was selected for subsequent immunizations.

**FIGURE 3:**
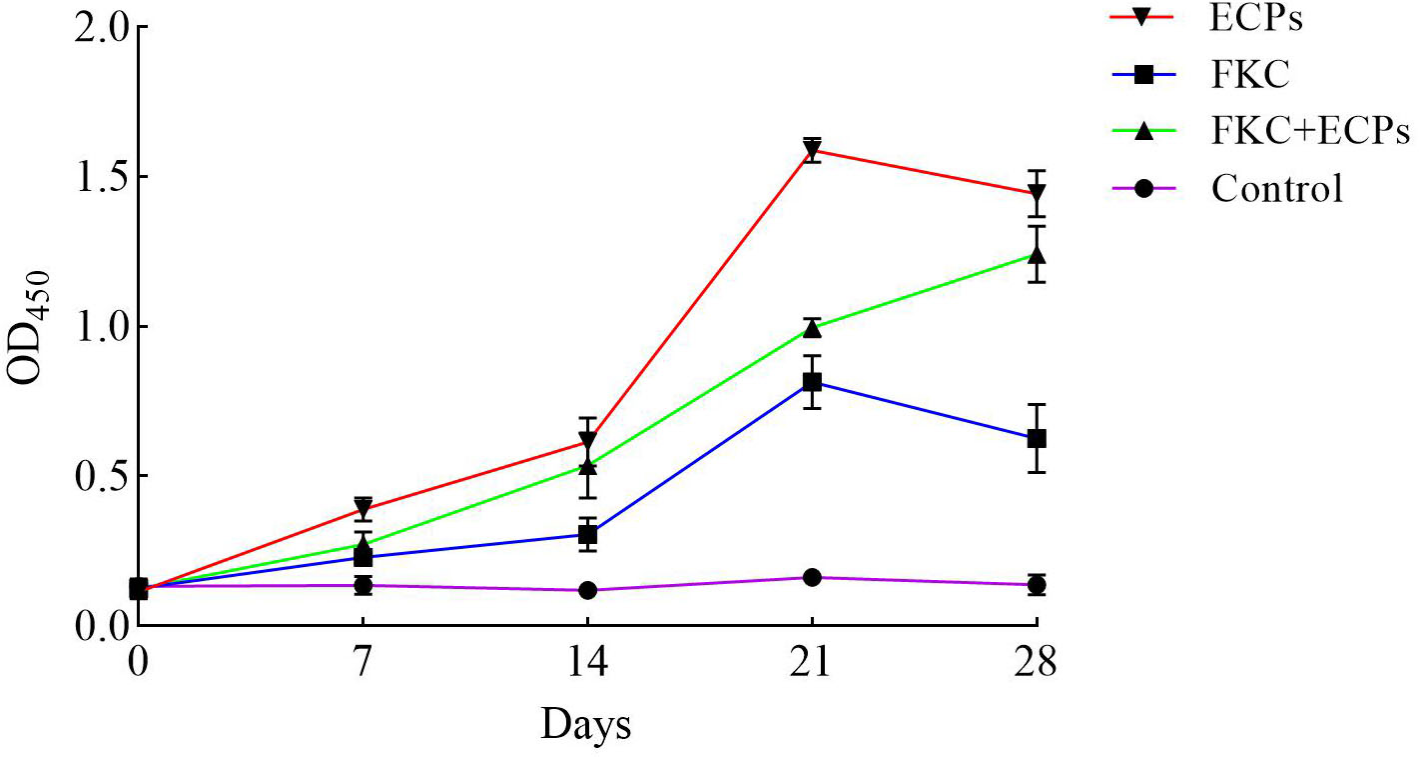
Determination of serum IgG levels in immunized mice. Mice were immunized with ECPs, FKC, or the combined FKC + ECPs vaccine. The whole-cell protein of PmQA-1 was used as the coating antigen, and serum IgG levels were measured by iELISA on days 0, 7, 14, 21, and 28 post-immunization. The PBS group received 0.2 mL of sterile PBS is the Control.

Serum samples were collected from the mice on days 0, 7, 14, 21, and 28 post-immunization with ECPs, and the levels of specific antibodies were measured using an indirect ELISA (iELISA). As shown in Figure 3, the levels of specific IgG in the sera of the ECPs and FKC immunization groups increased continuously, peaking on day 21. Although slightly decreased by day 28, the IgG levels remained significantly higher than those in the control group. In the FKC + ECPs immunization group, the specific IgG levels continued to rise throughout the experiment, reaching their maximum on day 28. Notably, the ECPs group exhibited higher specific IgG levels than the FKC + ECPs group, which in turn showed higher levels than the FKC group alone. All immunized groups demonstrated significantly higher specific IgG levels compared to the control group. These results indicate that ECPs induced the strongest humoral immune response, followed by the FKC + ECPs combination, while FKC alone elicited the weakest response.

### 3.5 Analysis of splenic lymphocyte proliferation in immunized mice

**FIGURE 4:**
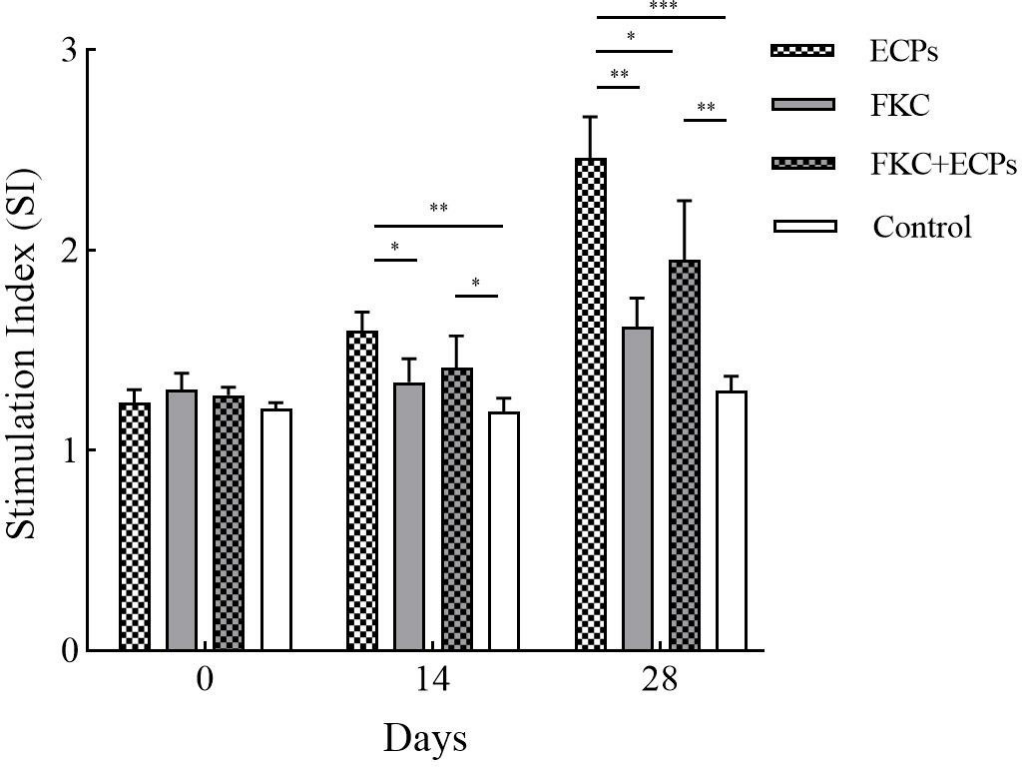
Splenic lymphocyte proliferation in immunized mice. Spleens were collected from three mice per group on days 0, 14, and 28 post-immunization. Splenic lymphocytes were isolated and adjusted to a concentration of 1 × 10^7^ CFU/mL. A total of 100 μL of cell suspension was added to each well of a 96-well plate, with PmQA-1 whole-cell protein added at a final concentration of 5 μg per well. Cells were incubated in a CO_2_ incubator for 48 h, and proliferation was assessed by measuring OD_570_ using the MTT assay. Statistical significance was indicated as follows: *p < 0.05, **p < 0.01, and ***p < 0.001.

Spleens were collected from mice on days 0, 14, and 28 after immunization with ECPs. The splenic lymphocyte proliferation index was determined using the MTT assay. As shown in Figure 4, the splenic lymphocyte proliferation levels in the ECPs, FKC, and FKC + ECPs immunization groups increased continuously, reaching their maximum on day 28, and were all significantly higher than those in the control group. Notably, the proliferation level in the ECPs group was higher than that in the FKC + ECPs group, which in turn was higher than that in the FKC group. These results indicate that ECPs induced the strongest cellular immune response, followed by the FKC + ECPs combination, while FKC alone elicited the weakest response.

### 3.6 Analysis of cytokine expression in organs of immunized mice

**FIGURE 5:**
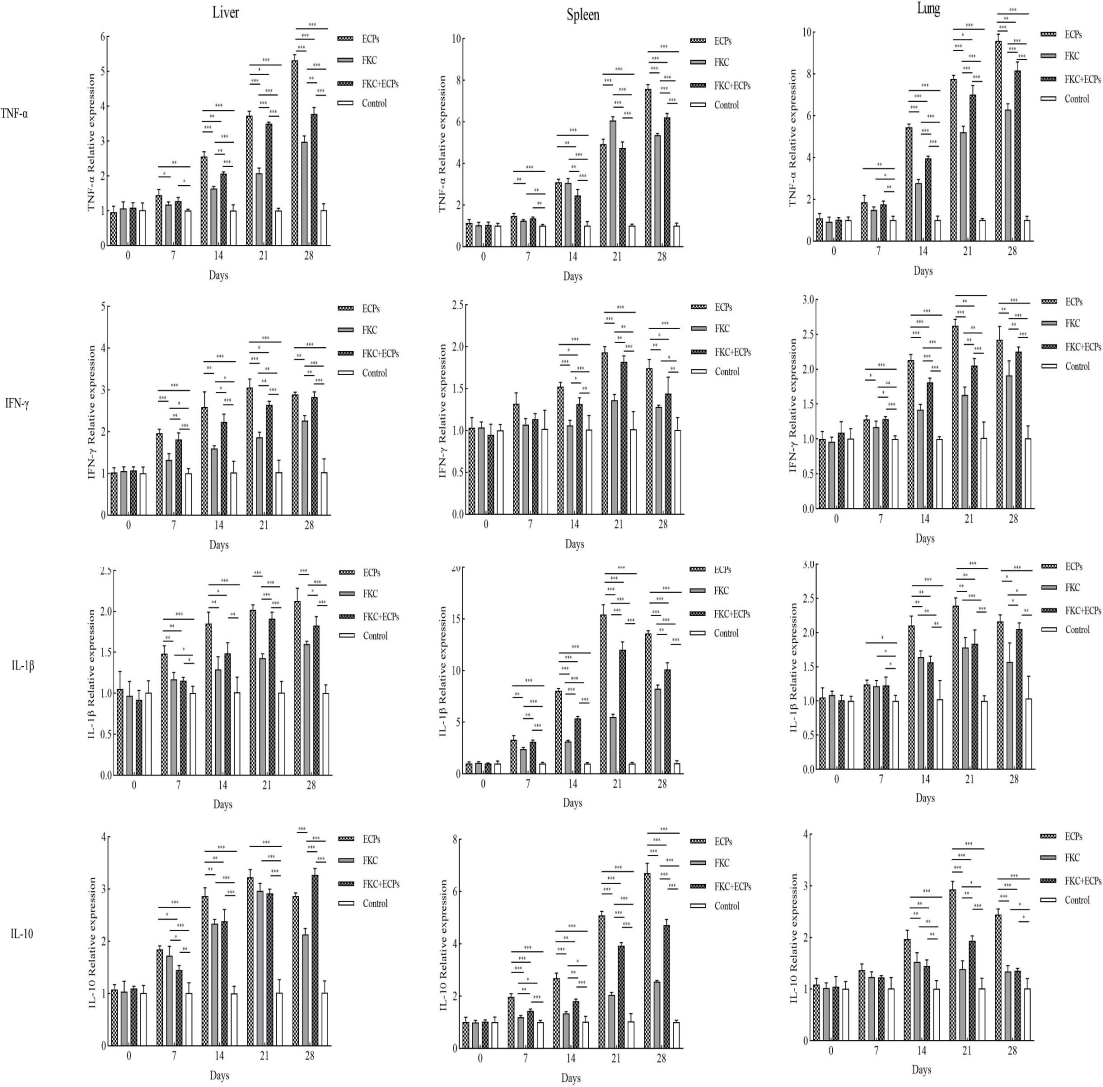
Expression levels of TNF-α, IFN-γ, IL-1β, and IL-10 in the liver, spleen, and lungs of mice immunized with ECPs, FKC, or FKC + ECPs. Cytokine expression was analyzed by qPCR using three mice per group. Data are presented as mean ± SD. Asterisks indicate significant differences compared to the negative control or among the experimental groups (*p < 0.05; **p < 0.01; ***p < 0.001).

The expression levels of TNF-α, IFN-γ, IL-1β, and IL-10 in the liver, spleen, and lungs of immunized mice were quantified by qPCR using GAPDH (Figure 5) and β-actin (Supplementary Figure 1) as reference genes. The results showed that in the ECPs, FKC, and FKC + ECPs groups, TNF-α expression in the liver, spleen, and lungs increased continuously, reaching peak levels on day 28, except in the spleen where TNF-α peaked on day 21. In the ECPs group, IFN-γ expression in the liver, spleen, and lungs increased steadily, peaking on day 21 and slightly declining on day 28. In the FKC and FKC + ECPs groups, splenic IFN-γ expression also peaked on day 21 and then slightly decreased, whereas hepatic and pulmonary IFN-γ expression continued to rise, reaching maximum levels on day 28. For IL-1β, expression in the liver of the ECPs group increased continuously and peaked on day 28, while expression in the spleen and lungs peaked on day 21 and slightly declined by day 28. In the FKC and FKC + ECPs groups, IL-1β expression in the liver, spleen, and lungs generally increased to a maximum on day 28, except for the lungs where it peaked on day 21 and decreased slightly afterward. Regarding IL-10, expression in the spleen of the ECPs group increased continuously, peaking on day 28. In the liver and lungs, IL-10 expression peaked on day 21 and slightly declined by day 28. In the FKC group, splenic IL-10 expression peaked on day 28, hepatic expression peaked on day 21 with a slight decline at day 28, and pulmonary IL-10 expression peaked earlier on day 14 and then declined. In the FKC + ECPs group, IL-10 expression in the liver and spleen increased continuously to reach peak levels on day 28, while in the lungs, it peaked on day 21 and then slightly decreased.

Notably, cytokine expression levels (TNF-α, IFN-γ, IL-1β, and IL-10) in the liver, spleen, and lungs were consistently higher in the ECPs group than in the FKC + ECPs group, which were in turn higher than those in the FKC group.

### 3.7 Protective efficacy of ECPs against infections with different *P. multocida* serotypes

**FIGURE 6:**
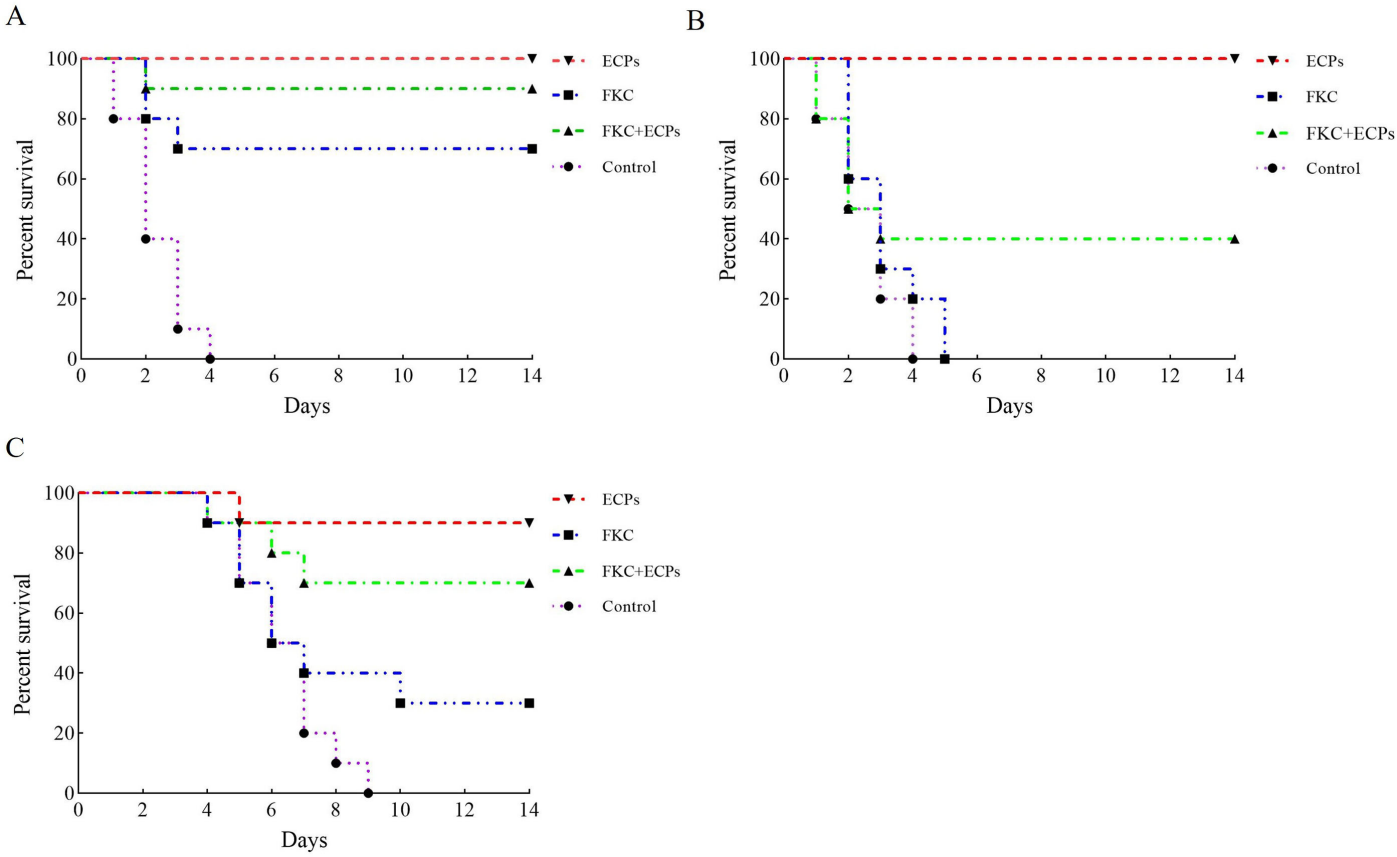
Protective efficacy of ECPs against different *P. multocida* serotypes in mice. Survival curves of mice subcutaneously immunized with extracellular products (ECPs), formalin-killed cells (FKC), or a combination of FKC + ECPs, or administered PBS as a control, and intraperitoneally challenged with *P. multocida* strains PmQA-1 **(A)**, PmQB-1 **(B)**, and PmQD-1 **(C)**.

At 28 days post-immunization, mice were challenged intraperitoneally with PmQA-1 (Figure 6A). All mice in the PBS control groups died following intraperitoneal challenge with any of the three serotypes. No deaths were observed in the ECPs-immunized group, resulting in a relative protection rate of 100%. In the FKC + ECPs group, only one mouse died on day 2 post-challenge, corresponding to a relative protection rate of 90%. In the FKC group, three mice died in total, yielding a relative protection rate of 70%. Following challenge with PmQB-1 (Figure 6B), the ECPs group again showed no mortality, maintaining a relative protection rate of 100%. In contrast, all mice in the FKC group died by day 5 post-challenge, resulting in a relative protection rate of 0%. In the FKC + ECPs group, six mice died in total, resulting in a protection rate of 40%. When challenged with PmQD-1 (Figure 6C), one mouse in the ECPs group died on day 5 post-challenge, giving a relative protection rate of 90%. Seven mice died in the FKC group, corresponding to a relative protection rate of 30%, while three deaths were recorded in the FKC + ECPs group, resulting in a protection rate of 70%.

These findings indicate that ECPs immunization provided the highest level of protection against all tested *P. multocida* serotypes, demonstrating its strong potential as a broad-spectrum vaccine candidate against *P. multocida* infections.

## 4 Discussion

Due to the high infection and mortality rates of *P. multocida*, vaccination is an important measure for preventing and controlling its infection. Previous studies have demonstrated that *P. multocida* serotype A is the predominant serotype isolated from cattle and poultry. However, the commercially available vaccines for bovine *P. multocida* are mainly developed based on serotype B. Due to the fact that the cross-immune protection effect among different serotypes of *P. multocida* is not satisfactory, the commercially available vaccines offer limited cross-protection against serotype A strains (Mostaan et al., 2020; Peng et al., 2016; Xiang et al., 2025). Extensive research has indicated that extracellular products (ECPs) from various pathogens contribute to bacterial pathogenicity and can also elicit strong immune responses in the host. In this study, we prepared ECPs from the *P. multocida* strain PmQA-1. Proteomic analysis revealed that the ECPs contained proteins ranging from 25 to 100 kDa, comprising at least 157 distinct proteins. Notably, these included Transferrin-binding protein A, Elongation factor Tu, Dihydrolipoyl dehydrogenase, Pyruvate dehydrogenase E1 component, Heme-binding protein A, MltA-interacting MipA protein, and Metalloprotease PmbA. Transferrin-binding protein A has been identified as a promising broad-spectrum vaccine candidate against *H. parasuis*, *Neisseria meningitidis*, and *P. multocida* serogroup B:2 (Fegan et al., 2019; Huang et al., 2013; Shivachandra et al., 2015; West et al., 2001). Similarly, Elongation factor Tu, Dihydrolipoyl dehydrogenase, Heme-binding protein, and MipA protein have all been reported to induce effective immune responses and confer protection against relevant pathogens (Devasundaram and Raja, 2017; Hays et al., 2016; Mou et al., 2020; Moustafa et al., 2024; Nagai et al., 2019; Thofte et al., 2018; Zhang et al., 2017). Additionally, studies have shown that ECPs derived from *Bacillus* spp., *V. lentus*, *Flavobacterium sasangense (F. sasangense)*, and *V. anguillarum* contain various immunogenic components capable of stimulating host immune responses and providing protection when used as vaccines (Chi et al., 2014; Santos et al., 1995; Song et al., 2018; Yuan et al., 2022). Based on these findings, we developed an ECP-based subunit vaccine and evaluated its protective efficacy against infections caused by different *P. multocida* serotypes.

Numerous studies have demonstrated that extracellular products (ECPs) carry various enzymes, including amylase, urease, protease, phospholipase, lipase, gelatinase, and hemolysin. These factors contribute to extensive tissue damage, facilitating nutrient acquisition and bacterial proliferation within the host, thereby playing a crucial role in pathogenicity (Fan et al., 2024; Zhang et al., 2014). Previous research has indicated that ECPs with proteolytic activity may suppress certain immune mechanisms by degrading immune components (Schadich and Cole, 2009; Song et al., 2018). In the present study, enzymatic activity assays revealed that the ECPs produced by PmQA-1 exhibited significant amylase activity. Amylase has been implicated as an important virulence factor in various pathogens. For example, loss of AMY1 function has been shown to reduce the ability of *Histoplasma* to kill macrophages and colonize murine lungs (Marion et al., 2006). Similarly, deletion of amyAXcc, a putative amylase-encoding gene in *Xanthomonas campestris* pv. *campestris* (*X. campestris* pv. *campestris*) 8004, resulted in complete loss of extracellular amylase activity and a marked reduction in virulence (Lin et al., 2021). To further verify the pathogenicity of ECPs, their median lethal dose (LD_50_) was determined by intraperitoneal injection in mice. Administration of ECPs caused mortality in mice, with necropsy revealing swelling and hemorrhage in the liver, spleen, and lungs, which closely resembled the pathological changes observed in mice challenged intraperitoneally with *P. multocida*. These findings confirm the significant role of ECPs in the pathogenicity of *P. multocida*.

Specific IgG levels in serum are a key indicator for evaluating the humoral immune response induced by vaccination, while the transformation rate of splenic lymphocytes can reflect the level of cellular immune response. In this study, both the serum IgG antibody levels and splenic lymphocyte transformation rates were significantly increased in mice immunized with ECPs, FKC, or the combination of FKC + ECPs. Notably, the ECPs-immunized group showed the highest IgG antibody titers and splenic lymphocyte transformation rates, indicating that the ECPs vaccine elicited the strongest humoral and cellular immune responses. Consistent with these findings, Zhang et al. (2014) reported that the agglutination titers of channel catfish immunized with the extracellular products (ECPs) of *A. hydrophila* increased from 2 to 4 weeks post-immunization and remained at a high level up to week seven. Similarly, Chi et al. (2014) demonstrated that the ECPs derived from three strains of intestinal autochthonous bacteria-*A. veronii* BA-1, *V. lentus* BA-2, and *F. sasangense* BA-3-significantly enhanced respiratory bursts, which are an important indicator of cellular immune mechanisms in fish, compared with the control group.

TNF-α is a pleiotropic cytokine that can activate macrophages and neutrophils to phagocytose pathogens and plays a key role in promoting inflammatory responses (Nguyen et al., 2016). IFN-γ is involved in Th1-type cellular immunity and induces cell-mediated immune responses (Kak et al., 2018). IL-1β is another important pro-inflammatory cytokine, while IL-10 participates in the regulation of cellular immunity and serves as a crucial anti-inflammatory cytokine (Weber et al., 2010). In this study, the relative expression levels of these cytokines in the liver, spleen, and lung tissues of immunized mice showed varying degrees of increase, reaching their peaks at either day 21 or day 28 post-immunization. Notably, the expression levels of cytokines in the ECPs-immunized group were higher than those in the FKC and FKC + ECPs groups across all examined tissues, indicating that ECPs immunization can elicit a stronger immune response and induce higher levels of cytokine production to protect the host. Similarly, Yuan et al. (2022) reported that crucian carp immunized with ECPs, FKC, or FKC + ECPs prepared from *A. caviae* AC-CY exhibited elevated cytokine levels in various tissues, with the ECPs group showing the most pronounced effect. This observation is consistent with the results of the present study, although the patterns of cytokine expression differed slightly.

To evaluate the protective efficacy of the vaccines, mice were intraperitoneally challenged with *P. multocida* strain PmQA-1. The results showed that immunization with ECPs conferred a relative protection rate of 100% against PmQA-1, providing robust homologous protection that was superior to that achieved by FKC + ECPs and FKC alone. These findings are consistent with previous studies on the protective effects of ECPs (Song et al., 2018; Zhang et al., 2014). Notably, the present study further demonstrated that ECPs conferred strong cross-protection, with protection rates of 100% and 87.5% against the heterologous strains PmQB-1 and PmQD-1, respectively. Similarly, Huang et al. (2013) reported that the TbpA protein, a major antigen presented in ECPs, provided cross-protection for guinea pigs against serovars 13, 4, and 14 of *G. parasuis*, for which at least 15 serovars have been identified. Given that *G. parasuis* and *P. multocida* are both members of the *Pasteurellaceae* family, the findings by Huang et al. (2013) and our study collectively indicate that ECPs and their key antigenic components hold considerable promise for the development of effective subunit vaccines with cross-serovar protection.

In summary, immunization of mice with ECPs derived from PmQA-1 significantly enhanced both humoral and cellular immune responses, increased cytokine expression levels, and provided effective cross-protection against the PmQA-1, PmQB-1, and PmQD-1 strains. These results highlight the potential of ECPs as a promising subunit vaccine candidate targeting multiple *P. multocida* serotypes. However, whether the protective efficacy and pathogenicity of ECPs are mediated by a single component or a combination of multiple components remains unclear, and further studies are needed to elucidate the underlying mechanisms and confirm their viability as vaccine candidates. In addition, it is necessary to extend the monitoring period for serum IgG levels, lymphocyte transformation rates, and cytokine expression to better understand the duration of the induced immune responses, thereby providing a more solid foundation for the development of effective subunit vaccines.

## 5 Conclusion

This study evaluated the immunoprotective efficacy of extracellular products (ECPs) from *Pasteurella multocida* serotype A:3 strain PmQA-1 in mice. ECPs contained 157 proteins (25–100 kDa), including transferrin-binding protein A and elongation factor Tu, with detectable amylase activity. ECPs exhibited pathogenicity (LD_50_ = 2.69 mg/mouse), inducing lesions consistent with *P. multocida* infection. ECPs vaccination triggered robust humoral immunity (peak IgG at day 21) and cellular responses (enhanced splenic lymphocyte proliferation). It also upregulated TNF-α, IFN-γ, IL-1β, and IL-10 in liver, spleen, and lungs, with peaks at days 21–28. Challenge tests showed ECPs conferred 100% protection against homologous serotype A:3 and heterologous serotype B:2, and 90% against serotype D:4, outperforming formalin-killed cell (FKC) vaccines and FKC + ECPs combinations. These results highlight ECPs as a promising broad-spectrum subunit vaccine candidate against *P. multocida* infections. One limitation of this study is that the immune protection experiments were conducted only in a murine infection model. Protective efficacy was not evaluated in livestock or poultry, which are the primary hosts of *P. multocida*. Further studies in these target animals are necessary to validate the applicability and effectiveness of the ECPs-based immunization strategy under practical field conditions.

## Data Availability

The raw data supporting the conclusions of this article will be made available by the authors, without undue reservation.

## References

[B1] Baldissera M. D. Souza C. F. Verdi C. M. Vizzotto B. S. Santos R. C. V. Baldisserotto B. (2018). Aeromonas caviae alters the activities of ecto-enzymes that hydrolyze adenine nucleotides in fish thrombocytes. *Microb. Pathog.* 115 64–67. 10.1016/j.micpath.2017.12.044 29253595

[B2] Chi C. Jiang B. Yu X. Liu T. Xia L. Wang G. (2014). Effects of three strains of intestinal autochthonous bacteria and their extracellular products on the immune response and disease resistance of common carp, cyprinus carpio. *Fish Shellfish Immunol.* 36 9–18. 10.1016/j.fsi.2013.10.003 24161775

[B3] Dabo S. M. Confer A. Montelongo M. York P. Wyckoff J. H. R. (2008). Vaccination with pasteurella multocida recombinant OmpA induces strong but non-protective and deleterious th2-type immune response in mice. *Vaccine* 26 4345–4351. 10.1016/j.vaccine.2008.06.029 18598730

[B4] Dassanayake R. P. Briggs R. E. Kaplan B. S. Menghwar H. Kanipe C. Casas E. (2025). Pasteurella multocida filamentous hemagglutinin b1 (fhab1) gene is not involved with avian fowl cholera pathogenesis in turkey poults. *BMC Vet. Res.* 21:207. 10.1186/s12917-025-04668-1 40140814 PMC11938644

[B5] Devasundaram S. Raja A. (2017). Dihydrolipoamide dehydrogenase-lpd (rv0462)-specific t cell recall responses are higher in healthy household contacts of TB: A novel immunodominant antigen from m. *Tuberculosis. J. Leukoc. Biol.* 102 135–151. 10.1189/jlb.4A0916-067RR 28428201

[B6] Fan C. Dai W. Zhang H. Liu S. Lin Z. Xue Q. (2024). Genomic and proteomic analyses of extracellular products reveal major virulence factors likely accounting for differences in pathogenicity to bivalves between vibrio mediterranei strains. *Animals* 14:692. 10.3390/ani14050692 38473077 PMC10930458

[B7] Fegan J. E. Calmettes C. Islam E. A. Ahn S. K. Chaudhuri S. Yu R. (2019). Utility of hybrid transferrin binding protein antigens for protection against pathogenic neisseria species. *Front. Immunol.* 10:247. 10.3389/fimmu.2019.00247 30837995 PMC6389628

[B8] Guan L. Zhang L. Xue Y. Yang J. Zhao Z. (2020). Molecular pathogenesis of the hyaluronic acid capsule of pasteurella multocida. *Microb. Pathog.* 149:104380. 10.1016/j.micpath.2020.104380 32645423

[B9] Gulliver E. L. Sy B. M. Wong J. L. Deveson Lucas D. S. Powell D. R. Harper M. (2022). The role and targets of the RNA-binding protein ProQ in the gram-negative bacterial pathogen pasteurella multocida. *J. Bacteriol.* 204:e0059221. 10.1128/jb.00592-21 35323048 PMC9017301

[B10] Hatfaludi T. Al-Hasani K. Gong L. Boyce J. D. Ford M. Wilkie I. W. (2012). Screening of 71 p. Multocida proteins for protective efficacy in a fowl cholera infection model and characterization of the protective antigen PlpE. *PLoS One* 7:e39973. 10.1371/journal.pone.0039973 22792202 PMC3390355

[B11] Hays M. P. Kumar A. Martinez-Becerra F. J. Hardwidge P. R. (2016). Immunization with the MipA, skp, or ETEC_2479 antigens confers protection against enterotoxigenic e. Coli strains expressing different colonization factors in a mouse pulmonary challenge model. *Front. Cell. Infect. Microbiol.* 6:181. 10.3389/fcimb.2016.00181 28018863 PMC5149512

[B12] Huang X. Li Y. Fu Y. Ji Y. Lian K. Zheng H. (2013). Cross-protective efficacy of recombinant transferrin-binding protein a of haemophilus parasuis in guinea pigs. *Clin. Vaccine Immunol.* 20 912–919. 10.1128/CVI.00621-12 23616407 PMC3675969

[B13] Kak G. Raza M. Tiwari B. K. (2018). Interferon-gamma (IFN-gamma): Exploring its implications in infectious diseases. *Biomol. Concepts* 9 64–79. 10.1515/bmc-2018-0007 29856726

[B14] Lee K. K. Chiang H. T. Yii K. C. Su W. M. Liu P. C. (1997). Effects of extracellular products of vibrio vulnificus on acanthopagrus schlegeli serum components in vitro and in vivo. *Microbios* 92 209–217. 9670552

[B15] Liao C. Huang C. Hsuan S. Chen Z. Lee W. Liu C. (2006). Immunogenicity and efficacy of three recombinant subunit pasteurella multocida toxin vaccines against progressive atrophic rhinitis in pigs. *Vaccine* 24 27–35. 10.1016/j.vaccine.2005.07.079 16122849

[B16] Lin Y. Liao Y. Huang R. Li A. An S. Tang J. (2021). Extracellular amylase is required for full virulence and regulated by the global posttranscriptional regulator RsmA in xanthomonas campestris pathovar campestris. *Phytopathology* 111 1104–1113. 10.1094/PHYTO-08-20-0372-R 33245253

[B17] MacKinnon B. Groman D. Fast M. D. Manning A. J. Jones P. St-Hilaire S. (2019). Atlantic salmon challenged with extracellular products from moritella viscosa. *Dis. Aquat. Org.* 133 119–125. 10.3354/dao03337 31019136

[B18] Marion C. L. Rappleye C. A. Engle J. T. Goldman W. E. (2006). An alpha-(1,4)-amylase is essential for alpha-(1,3)-glucan production and virulence in histoplasma capsulatum. *Mol. Microbiol.* 62 970–983. 10.1111/j.1365-2958.2006.05436.x 17038119

[B19] Megroz M. Kleifeld O. Wright A. Powell D. Harrison P. Adler B. (2016). The RNA-binding chaperone hfq is an important global regulator of gene expression in pasteurella multocida and plays a crucial role in production of a number of virulence factors, including hyaluronic acid capsule. *Infect. Immun.* 84 1361–1370. 10.1128/IAI.00122-16 26883595 PMC4862696

[B20] Mostaan S. Ghasemzadeh A. Sardari S. Shokrgozar M. A. Nikbakht Brujeni G. Abolhassani M. (2020). Pasteurella multocida vaccine candidates: A systematic review. *Avicenna J. Med. Biotechnol.* 12 140–147. 32695276 PMC7368114

[B21] Mou Z. Barazandeh A. F. Hamana H. Kishi H. Zhang X. Jia P. (2020). Identification of a protective leishmania antigen dihydrolipoyl dehydrogenase and its responding CD4(+) t cells at clonal level. *J. Immunol.* 205 1355–1364. 10.4049/jimmunol.2000338 32727889

[B22] Moustafa D. A. Lou E. Schafer-Kestenman M. E. Mateu-Borras M. Domenech-Sanchez A. Alberti S. (2024). *Pseudomonas aeruginosa* elongation factor-tu (EF-tu) is an immunogenic protective protein antigen. *Vaccine* 42:126476. 10.1016/j.vaccine.2024.126476 39476472 PMC11645190

[B23] Nagai K. Domon H. Maekawa T. Hiyoshi T. Tamura H. Yonezawa D. (2019). Immunization with pneumococcal elongation factor tu enhances serotype-independent protection against streptococcus pneumoniae infection. *Vaccine* 37 160–168. 10.1016/j.vaccine.2018.11.015 30442480

[B24] Nguyen Q. H. Lai C. H. R. Norris M. J. Ng D. Shah M. Lai C. C. (2025). A surface lipoprotein on pasteurella multocida binds complement factor i to promote immune evasion. *PLoS Pathog.* 21:e1012686. 10.1371/journal.ppat.1012686 40327719 PMC12080921

[B25] Nguyen T. H. Maltby S. Simpson J. L. Eyers F. Baines K. J. Gibson P. G. (2016). TNF-alpha and macrophages are critical for respiratory syncytial virus-induced exacerbations in a mouse model of allergic airways disease. *J. Immunol.* 196 3547–3558. 10.4049/jimmunol.1502339 27036916

[B26] Okay S. Ozcengiz E. Gursel I. Ozcengiz G. (2012). Immunogenicity and protective efficacy of the recombinant pasteurella lipoprotein e and outer membrane protein h from pasteurella multocida a:3 in mice. *Res. Vet. Sci.* 93 1261–1265. 10.1016/j.rvsc.2012.05.011 22727197

[B27] Pemberton J. M. Kidd S. P. Schmidt R. (1997). Secreted enzymes of aeromonas. *FEMS Microbiol. Lett.* 152 1–10. 10.1111/j.1574-6968.1997.tb10401.x 9228763

[B28] Peng Z. Liang W. Liu W. Wu B. Tang B. Tan C. (2016). Genomic characterization of pasteurella multocida HB01, a serotype a bovine isolate from china. *Gene* 581 85–93. 10.1016/j.gene.2016.01.041 26827796

[B29] Pettit R. K. Rimler R. B. Ackermann M. R. (1993). Protection of pasteurella multocida dermonecrotic toxin-challenged rats by toxoid-induced antibody. *Vet. Microbiol.* 34 167–173. 10.1016/0378-1135(93)90170-c 8451832

[B30] Piorunek M. Brajer-Luftmann B. Walkowiak J. (2023). Pasteurella multocida infection in humans. *Pathogens* 12:1210. 10.3390/pathogens12101210 37887726 PMC10610061

[B31] Ryan J. M. Feder H. M. J. (2019). Dog licks baby. Baby gets pasteurella multocida meningitis. *Lancet* 393:e41. 10.1016/S0140-6736(19)30953-5 31226054

[B32] Sahu I. Das B. K. Marhual N. Samanta M. Mishra B. K. Eknath A. E. (2011). Toxicity of crude extracellular products of aeromonas hydrophila on rohu, labeo rohita (ham.). *Indian J. Microbiol.* 51 515–520. 10.1007/s12088-011-0182-6 23024416 PMC3209931

[B33] Santos Y. Pazos F. Bandin I. Toranzo A. E. (1995). Analysis of antigens present in the extracellular products and cell surface of vibrio anguillarum serotypes o1, o2, and o3. *Appl. Environ. Microbiol.* 61 2493–2498. 10.1128/aem.61.7.2493-2498.1995 7618861 PMC167521

[B34] Schadich E. Cole A. L. J. (2009). Inhibition of frog antimicrobial peptides by extracellular products of the bacterial pathogen aeromonas hydrophila. *Lett. Appl. Microbiol.* 49 384–387. 10.1111/j.1472-765x.2009.02677.x 19728406

[B35] Shivachandra S. B. Yogisharadhya R. Kumar A. Mohanty N. N. Nagaleekar V. K. (2015). Recombinant transferrin binding protein a (rTbpA) fragments of pasteurella multocida serogroup b:2 provide variable protection following homologous challenge in mouse model. *Res. Vet. Sci.* 98 1–6. 10.1016/j.rvsc.2014.11.013 25544697

[B36] Song M. Kang Y. Zhang D. Chen L. Bi J. Zhang H. (2018). Immunogenicity of extracellular products from an inactivated vaccine against aeromonas veronii TH0426 in koi, cyprinus carpio. *Fish Shellfish Immunol.* 81 176–181. 10.1016/j.fsi.2018.07.004 30026173

[B37] Sun Q. Yu X. He D. Ku X. Hong B. Zeng W. (2022). Investigation and analysis of etiology associated with porcine respiratory disease complex in china from 2017 to 2021. *Front. Vet. Sci.* 9:960033. 10.3389/fvets.2022.960033 36304408 PMC9592729

[B38] Thofte O. Su Y. Brant M. Littorin N. Duell B. L. Alvarado V. (2018). EF-tu from non-typeable *haemophilus influenzae* is an immunogenic surface-exposed protein targeted by bactericidal antibodies. *Front. Immunol.* 9:2910. 10.3389/fimmu.2018.02910 30619274 PMC6305414

[B39] Varshney R. Varshney R. Chaturvedi V. K. Rawat M. Saminathan M. Singh V. (2020). Development of novel iron-regulated pasteurella multocida b: 2 bacterin and refinement of vaccine quality in terms of minimum variation in particle size and distribution vis-a-vis critical level of iron in media. *Microb. Pathog.* 147:104375. 10.1016/j.micpath.2020.104375 32679244

[B40] Weber A. Wasiliew P. Kracht M. (2010). Interleukin-1beta (IL-1beta) processing pathway. *Sci. Signal.* 3:cm2. 10.1126/scisignal.3105cm2 20086236

[B41] West D. Reddin K. Matheson M. Heath R. Funnell S. Hudson M. (2001). Recombinant *neisseria meningitidis* transferrin binding protein a protects against experimental meningococcal infection. *Infect. Immun.* 69 1561–1567. 10.1128/IAI.69.3.1561-1567.2001 11179327 PMC98056

[B42] Wilkie I. W. Harper M. Boyce J. D. Adler B. (2012). Pasteurella multocida: Diseases and pathogenesis. *Curr. Top. Microbiol. Immunol.* 361 1–22. 10.1007/82_2012_216 22643916

[B43] Xiang X. Sun Y. Zhang H. Wu X. Xia J. Han X. (2025). Evaluation of immunoprotective effects of PlpE multi-epitope protein incorporated within the aluminum hydroxide-adjuvanted inactivated vaccine against pasteurella multocida infection in chickens. *Poult. Sci.* 104:105426. 10.1016/j.psj.2025.105426 40561824 PMC12241968

[B44] Yuan Z. Song H. Huang Q. Liu J. Sun H. Meng X. (2022). Immune enhancement effects of inactivated vaccine against extracellular products of aeromonas caviae AC-CY on crucian carp. *Fish Shellfish Immunol.* 127 1001–1011. 10.1016/j.fsi.2022.07.046 35870745

[B45] Zhan L. Zhang J. Zhao B. Li X. Zhang X. Hu R. (2021). Genomic and transcriptomic analysis of bovine pasteurella multocida serogroup a strain reveals insights into virulence attenuation. *Front. Vet. Sci.* 8:765495. 10.3389/fvets.2021.765495 34859092 PMC8631534

[B46] Zhang D. Pridgeon J. W. Klesius P. H. (2014). Vaccination of channel catfish with extracellular products of aeromonas hydrophila provides protection against infection by the pathogen. *Fish Shellfish Immunol.* 36 270–275. 10.1016/j.fsi.2013.11.015 24321514

[B47] Zhang X. Song Y. Li Y. Cai M. Meng Y. Zhu H. (2017). Immunization with streptococcal heme binding protein (shp) protects mice against group a streptococcus infection. *Adv. Exp. Med. Biol.* 973 115–124. 10.1007/5584_2016_198 28190144

[B48] Zhao G. Tang Y. Liu X. Li P. Zhang T. Li N. (2024). Pasteurella multocida activates rassf1-hippo-yap pathway to induce pulmonary epithelial apoptosis. *Vet. Res.* 55:31. 10.1186/s13567-024-01285-y 38493147 PMC10943858

[B49] Zhao X. Yang F. Shen H. Liao Y. Zhu D. Wang M. (2022). Immunogenicity and protection of a pasteurella multocida strain with a truncated lipopolysaccharide outer core in ducks. *Vet. Res.* 53:17. 10.1186/s13567-022-01035-y 35236414 PMC8889768

[B50] Zhao Y. Liu Y. Li N. Muhammad M. Gong S. Ju J. (2022). Significance of broad-spectrum racemases for the viability and pathogenicity of aeromonas hydrophila. *Future Microbiol.* 17 251–265. 10.2217/fmb-2021-0112 35152710

