# Supplementary material for: Immunoprotective effects of extracellular products of Pasteurella multocida on mice
Source: Front Microbiol. 2025 Sep 9;16:1674831. doi: 10.3389/fmicb.2025.1674831 (PMC12454335; doi:10.3389/fmicb.2025.1674831)
Supplement: Supplementary file 1 [file Data_Sheet_1.docx]

**Table S1** LD_50_ of PmQA-1, PmQB-1, PmQD-1 and ECPs in KM mice

| Group | Inoculation dose  (CFU/mouse) | No.of deaths/ total no. of mice | LD_50_  (CFU/mouse) |
| --- | --- | --- | --- |
| PmQA-1 | 3.9×10^4^ | 5/5 | 1.55×10^1^ |
|  | 3.9×10^3^ | 5/5 |  |
|  | 3.9×10^2^ | 4/5 |  |
|  | 3.9×10^1^ | 3/5 |  |
|  | 3.9×10^0^ | 2/5 |  |
| PmQB-1 | 9.73×10^4^ | 5/5 | 8.67 |
|  | 9.73×10^3^ | 5/5 |  |
|  | 9.73×10^2^ | 5/5 |  |
|  | 9.73×10^1^ | 4/5 |  |
|  | 9.73×10^0^ | 3/5 |  |
| PmQD-1 | 2.47×10^4^ | 4/5 | 8.76×10^2^ |
|  | 2.47×10^3^ | 3/5 |  |
|  | 2.47×10^2^ | 2/5 |  |
|  | 2.47×10^1^ | 1/5 |  |
|  | 2.47×10^0^ | 0/5 |  |
| PBS | 0 | 0/5 |  |
| Group | Inoculation dose  (mg/mouse) | No.of deaths/ total no. of mice | LD_50_  (mg/mouse) |
| ECPs | 4.08 | 3/5 | 2.69 |
|  | 2.04 | 2/5 |  |
|  | 1.02 | 1/5 |  |
|  | 0.51 | 0/5 |  |
|  | 0.26 | 0/5 |  |
| PBS | 0 | 0/5 |  |
