# Supplementary figures and images for: Immunoprotective effects of extracellular products of Pasteurella multocida on mice
Source: Front Microbiol. 2025 Sep 9;16:1674831. doi: 10.3389/fmicb.2025.1674831 (PMC12454335; doi:10.3389/fmicb.2025.1674831)

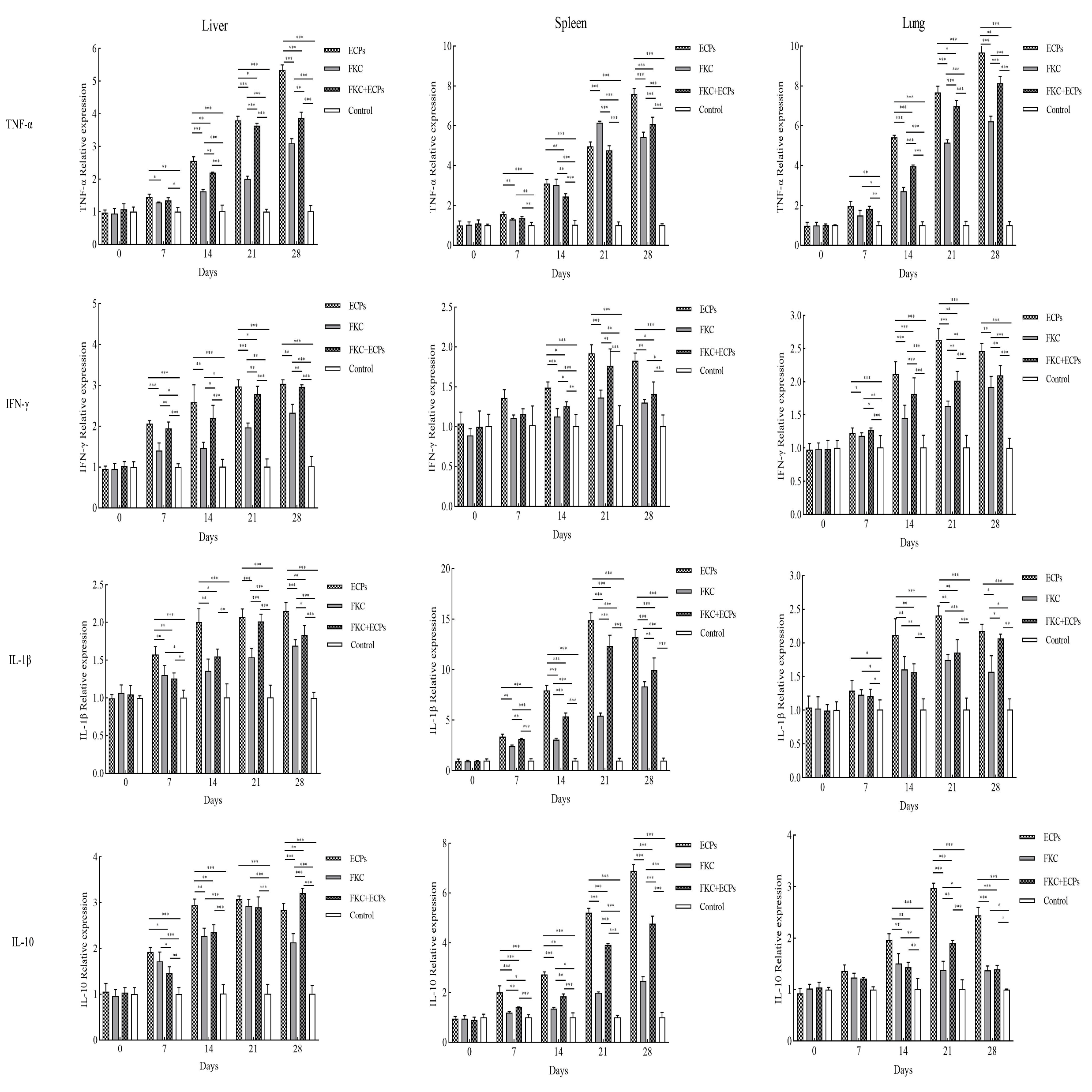

Supplement: Supplementary file 2 [file Image_1.tif]
